# Supplementary material for: Elzaki residual power series method to solve fractional diffusion equation
Source: PLoS One. 2024 Mar 20;19(3):e0298064. doi: 10.1371/journal.pone.0298064 (PMC10954171; doi:10.1371/journal.pone.0298064)
Supplement: Attachment — Submitted filename: Review reports.pdf [file pone.0298064.s003.pdf]

Journal title: PLOS ONE
Subject: Review reports

After reading the revised manuscript carefully, I found that some of the questions raised by the previous reviewers has not been answered correctly. As a result, I suggest the following corrections before accepting this manuscript.

1. Define the Caputo fractional derivative, the Mittag-Leffler function, and the Elzaki transform of Caputo fractional derivative in the preliminary section.

2. The authors claim that $E(D_t^\alpha u(x,y,t)) = \frac{1}{v^\alpha}\left(E(u(x,y,t)) - (x,y,t)\right).$ However, this is not correct, since

$$E(D_t^\alpha u(x,y,t)) = \frac{1}{v^\alpha}E(u(x,y,t)) - \sum_{i=0}^{m-1} v^{2+i-\alpha}\frac{\partial^k u(x,y,t)}{\partial t^k}\mid_{t=0},\ m-1<\alpha<m. \tag{1}$$

Based on equation (1) above, the correct version of equation (5) is:

$$\frac{1}{u^\alpha}E(u(x,y,t)) - \sum_{i=0}^{m-1} u^{2+i-\alpha}\frac{\partial^k u(x,y,t)}{\partial t^k}\mid_{t=0} = E(u_{xx}(x,y,t)+u_{yy}(x,y,t))$$
$$E(u(x,y,t)) = v^2u(x,y,0) + v^\alpha\left(E(u_{xx}(x,y,t)+u_{yy}(x,y,t))\right). \tag{2}$$

Therefore, the authors must modify section 3 and section 4 carefully as suggested in the first review.
