# Supplementary material for: Elzaki residual power series method to solve fractional diffusion equation
Source: PLoS One. 2024 Mar 20;19(3):e0298064. doi: 10.1371/journal.pone.0298064 (PMC10954171; doi:10.1371/journal.pone.0298064)
Supplement: Attachment — Submitted filename: Response to Reviewer-Plos one.docx [file pone.0298064.s002.docx]

Journal title: PLOS ONE

Subject: Review reports

In this paper, the author proposed a powerful analytical method for solving two-dimensional fractional order diffusion equation called the residual power series method (RPS) via Elzaki transform method. The fractional derivative is computed in Caputo sense. The proposed analytical method is successfully applied to two-dimensional fractional order diffusion equation and the numerical solutions for different values of α are provided. The 3D graphical solutions of the two-dimensional fractional order diffusion equation are provided. In general, the author introduce a very interesting topic and the novelty of this manuscript is remarkable and deserve to be published after the following minor revision.

1. The abstract of the manuscript should be carefully modified. For example the letters ”RPS” should be written in full in the abstract.

RPS is replaced by residual power series and the abstract is reframed.

2. Improved the introduction section of the manuscript with the latest papers in the topic.

Introduction is updated.

3. After modifying the introduction section, create preliminary section which contains the basic definitions of fractional derivatives and integral used in this manuscript.

Preliminaries are updated

4. The derivation in the methodology section is not correct. For example, in eq(5) there is a term g(x, y, t) while in eq(4) which is the original problem the term g(x, y, t) is missing. The authors must carefully modified the methodology section.

As per the differentiation property of Elzaki transforms,

${E[D}_{t}^{\alpha}u(x,y,t)]=\frac{1}{\upsilon^{\alpha}}\{E\left( u\left( x,y,t \right) \right)-g(x,y,t)\}$ is used on equation (4), so there is no change in $g(x,y,t)$ in equation (5), it will remains as it is.

5. In the methodology section, the authors should used a more general equation rather than a particular problem. For example, the authors refer to section 2 in [1].

Methodology section is updated as standard papers as previous.

6. The fond size in the methodology section are not the same. I advice the authors to adopt a uniform fond size using proper mathematical tools. For example using Latex or any other mathematical tool.

Font size are made as same as possible.

7. The authors should prove the convergence analysis and error estimate of the proposed method.

Elzaki transform is used for finite number of terms hence the method is convergent. The convergence of RPS is discussed with help of theorem from literature in the preliminary section.

8. Figure 1 The pseudo code of the methodology is not clear. The authors should provide a clear figure.

Pseudo code of the methodology is updated.

9. The numerical solution section (section 3) is a mire repetition of the methodology section (section 2). Besides,the term g(x, y, t) which appears in eq(12) is missing in eq(11). I advice the authors to change the application procedure to modified the application section.

Using the differentiation property of Elzaki transform,

${E[D}_{t}^{\alpha}u(x,y,t)]=\frac{1}{\upsilon^{\alpha}}\{E\left( u\left( x,y,t \right) \right)-g(x,y,t)\}$ on equation (11) to get equation (12), so the term $g(x,y,t)$ comes from this property on ${E[D}_{t}^{\alpha}u(x,y,t)]$.

10. The fond size in section 3 are not the same. I advice the authors to adopt a uniform fond size using proper mathematical tools. For example using Latex or any other mathematical tool.

Font size is adjusted.

11. The authors should apply the proposed method to nonlinear two-dimensional fractional order diffusion equation in section 3.

In section 3 the methodology has been discussed and the method is applied in section 4 to an example.

12. Create a results and discussion section after section 3.

The results and discussions are given in section 5 after the implementation of method of numerical example.

13. In Table 1, Table 2, Table 3, and Table 4 write alpha as α.

Updated as suggested.

14. Please, format the manuscript according to the journal template.

Updated according as journal template.

15. I found no comparative results within this manuscript. Please, provide some comparative results with the other methods in results and discussion section.

The results are compared in Table 5.

16. The language of the manuscript should be modified carefully.

Language of the manuscript is updated.

17. The authors should outline the advantage of using the Elzaki transform rather than the Laplace transform or any other integral transforms in the literature.

Elzaki transform is modified form of Laplace transform and Sumudu transform so it is definitely advantageous. Discussed in introduction section.

18. Modified the conclusion section of the manuscript.

Conclusion section is also updated.

19. Please, format the manuscript according to the journal template.

Updated as suggested.

20. The references of this manuscript should be modified with the recent developments in fractional calculus as well as its applications.

Updated as required.
