# Supplementary material for: Elzaki residual power series method to solve fractional diffusion equation
Source: PLoS One. 2024 Mar 20;19(3):e0298064. doi: 10.1371/journal.pone.0298064 (PMC10954171; doi:10.1371/journal.pone.0298064)
Supplement: Attachment — Submitted filename: Responses to Review reports.docx [file pone.0298064.s004.docx]

**Journal title**: PLOS ONE

**Subject:** Review reports

After reading the revised manuscript carefully, I found that some of the questions raised by the previous reviewers has not been answered correct- ly. As a result, I suggest the following corrections before accepting this manuscript.

1. Define the Caputo fractional derivative, the Mittag-Leffler function, and the Elzaki transform of Caputo fractional derivative in the pre- liminary section.

Updated as suggested.

2. The authors claim that *E*(*D^α^u*(*x, y, t*)) = ^1^ (*E*(*u*(*x, y, t*)) *−* (*x, y, t*)) *.*

*t	vα*

However, this is not correct, since

*E*(*D^α^u*(*x, y, t*)) = 1 *E*(*u*(*x, y, t*))*−* Σ *v*^2+^*^i−α^ ∂ u*(*x, y, t*) *|*

*m−*1	*k*

*, m−*1 *< α < m.*

*t	vα*

*i*=0

*∂tk*

*t*=0

(1)

Based on equation (1) above, the correct version of equation (5) is:

*m−*1	*k*

1 *E*(*u*(*x, y, t*)) *−* Σ *u*^2+^*^i−α^ ∂ u*(*x, y, t*) *|*	= *E*(*u*	(*x, y, t*) + *u*	(*x, y, t*))

*i*=0

*uα*

*∂tk*

*t*=0

*xx*

*yy*

*E*(*u*(*x, y, t*)) = *v*^2^*u*(*x, y,* 0) + *v^α^* (*E*(*u_xx_*(*x, y, t*) + *u_yy_*(*x, y, t*))) *.*	(2)

Therefore, the authors must modify section 3 and section 4 carefully as suggested in the first review.

Updated as required as suggested with right result of Elzaki transform.
